# Supplementary material for: Costs of outpatient services at selected primary healthcare centers in Bangladesh: A cross-sectional study
Source: PLoS One. 2025 Jan 14;20(1):e0317317. doi: 10.1371/journal.pone.0317317 (PMC11731759; doi:10.1371/journal.pone.0317317)
Supplement: Attachment — Submitted filename: PONE-D-24-19534.pdf [file pone.0317317.s006.pdf]

PLOS ONE

Costs of outpatient services at selected primary healthcare centers in rural Bangladesh: a cross-sectional study

--Manuscript Draft--

**Manuscript Number:**	PONE-D-24-19534
**Article Type:**	Research Article
**Full Title:**	Costs of outpatient services at selected primary healthcare centers in rural Bangladesh: a cross-sectional study
**Short Title:**	Costs of outpatient services at primary healthcare centers in Bangladesh
**Corresponding Author:**	Md. Zahid Hasan<br>ICDDRB: International Centre for Diarrhoeal Disease Research Bangladesh<br>Dhaka, Dhaka District BANGLADESH
**Keywords:**	primary healthcare; Outpatient services; Out-of-pocket expenditure; Societal cost; Bangladesh
**Abstract:**	Background: Upazila Health Complexes (UzHC) serve as the backbone of primary healthcare at sub-district level in rural Bangladesh, delivering comprehensive healthcare services to the grassroots level. However, not all the prescribed medicines and diagnostics services are always available at these facilities for outpatient care. This results in out-of-pocket expenditure (OOPE) to the patients for getting the prescribed medicines and diagnostics services which is not properly explored. Thus, we aimed to estimate the overall provider and user costs for outpatient care services at selected UzHCs in Bangladesh.<br>Methods: An ingredient-based costing approach was applied to estimate the costs for the most commonly reported illnesses at outpatient from societal perspective. A health facility survey at four purposively selected UzHCs to estimate provider costs and a patient exit survey among 452 patients of selected illness to estimate the user costs. We identified commonly reported illnesses in consultation with healthcare providers of these facilities. The difference between costs for prescribed and provided medicines at UzHCs were estimated using the market prices. Data was collected between February to March 2021.<br>Results: The societal cost of the common outpatient illness or symptoms varied significantly, ranging from BDT 642 to BDT 1,384 per episode. Antenatal care topped the list with the highest cost burden at BDT 1,384, followed by BDT 783 for Respiratory illness and BDT 670 for Urinary Tract Infections. On average the provider spent BDT 289 for treating an outpatient, while a patient incurred BDT 446 as OOPE. On an average a patient were expected to spent BDT 341 for purchasing medicines those were not provided from UzHCs.<br>Conclusion: Our study found significant gaps between prescribed and provided medicines at UzHCs, leading to higher OOPE for patients. This might result into non-compliance to prescriptions for the low-income patients increasing risks of poor health outcomes. The current healthcare resource allocation strategy does not consider the outpatient load and healthcare demand, which further exacerbates this gap. Addressing this gap requires a fundamental shift towards a demand-driven resource allocation model in the healthcare financing strategy to ensure better treatment compliance and achieve health for all.
**Order of Authors:**	Md. Zahid Hasan
	Gazi Goalm Mehdi
	Khadija Islam Islam Tisha
	Md Golam Rabbani
	Mohammad Wahid Ahmed
	Subrata Paul
	Ziaul Islam
	Shehrin Shaila Mahmood

Powered by Editorial Manager® and ProduXion Manager® from Aries Systems Corporation

Opposed Reviewers:	
Additional Information:	
**Question**	**Response**
**Financial Disclosure**<br><br>Enter a financial disclosure statement that describes the sources of funding for the work included in this submission. Review the submission guidelines for detailed requirements. View published research articles from *PLOS ONE* for specific examples.<br><br>This statement is required for submission and **will appear in the published article** if the submission is accepted. Please make sure it is accurate.<br><br>**Funded studies**<br>Enter a statement with the following details:<br>• Initials of the authors who received each award<br>• Grant numbers awarded to each author<br>• The full name of each funder<br>• URL of each funder website<br>• Did the sponsors or funders play any role in the study design, data collection and analysis, decision to publish, or preparation of the manuscript?<br><br>Did you receive funding for this work?	Yes
Please add funding details.<br>as follow-up to "**Financial Disclosure**<br><br>Enter a financial disclosure statement that describes the sources of funding for the work included in this submission. Review the submission guidelines for detailed requirements. View published research articles from *PLOS ONE* for specific examples.<br><br>This statement is required for submission and **will appear in the published article** if the submission is accepted. Please make sure it is accurate.	The study was funded by the Health Economics Unit (HEU), Health Services Division, Ministry of Health and Family Welfare, the Government of Bangladesh (Grant #: GR-01964).

**Funded studies**<br>Enter a statement with the following details:<br>• Initials of the authors who received each award<br>• Grant numbers awarded to each author<br>• The full name of each funder<br>• URL of each funder website<br>• Did the sponsors or funders play any role in the study design, data collection and analysis, decision to publish, or preparation of the manuscript?<br><br>Did you receive funding for this work?"	
Please select the country of your main research funder (please select carefully as in some cases this is used in fee calculation).<br>as follow-up to "**Financial Disclosure**<br><br>Enter a financial disclosure statement that describes the sources of funding for the work included in this submission. Review the submission guidelines for detailed requirements. View published research articles from *PLOS ONE* for specific examples.<br><br>This statement is required for submission and **will appear in the published article** if the submission is accepted. Please make sure it is accurate.<br><br>**Funded studies**<br>Enter a statement with the following details:<br>• Initials of the authors who received each award<br>• Grant numbers awarded to each author<br>• The full name of each funder<br>• URL of each funder website<br>• Did the sponsors or funders play any role in the study design, data collection and analysis, decision to publish, or preparation of the manuscript?<br><br>Did you receive funding for this work?"	BANGLADESH - BD
**Competing Interests**	The authors have declared that no competing interests exist.

Use the instructions below to enter a competing interest statement for this submission. On behalf of all authors, disclose any competing interests that could be perceived to bias this work—acknowledging all financial support and any other relevant financial or non-financial competing interests.<br><br>This statement is required for submission and **will appear in the published article** if the submission is accepted. Please make sure it is accurate and that any funding sources listed in your Funding Information later in the submission form are also declared in your Financial Disclosure statement.<br><br>View published research articles from *PLOS ONE* for specific examples.<br><br>**NO authors have competing interests**<br><br>Enter: *The authors have declared that no competing interests exist.*<br><br>**Authors with competing interests**<br><br>Enter competing interest details beginning with this statement:<br><br>*I have read the journal's policy and the authors of this manuscript have the following competing interests: [insert competing interests here]*<br><br>* typeset	
**Ethics Statement**<br><br>Enter an ethics statement for this submission. This statement is required if the study involved:<br><br>• Human participants<br>• Human specimens or tissue<br>• Vertebrate animals or cephalopods<br>• Vertebrate embryos or tissues	Ethical approval for the study was obtained from the institutional review board of the International Centre for Diarrhoeal Disease Research, Bangladesh (icddr,b) before data collection (PR#20144). Informed written consent was taken from all the respondents prior to the interviews, and confidentiality and anonymity of their provided information were ensured. All data were collected anonymously and handled confidentially, aligning with the declaration of Helsinki.

• Field research<br><br>Write "N/A" if the submission does not require an ethics statement.<br><br>General guidance is provided below. Consult the submission guidelines for detailed instructions. **Make sure that all information entered here is included in the Methods section of the manuscript.**	

Powered by Editorial Manager® and ProduXion Manager® from Aries Systems Corporation

**Format for specific study types**<br><br>**Human Subject Research (involving human participants and/or tissue)**<br>• Give the name of the institutional review board or ethics committee that approved the study<br>• Include the approval number and/or a statement indicating approval of this research<br>• Indicate the form of consent obtained (written/oral) or the reason that consent was not obtained (e.g. the data were analyzed anonymously)<br><br>**Animal Research (involving vertebrate animals, embryos or tissues)**<br>• Provide the name of the Institutional Animal Care and Use Committee (IACUC) or other relevant ethics board that reviewed the study protocol, and indicate whether they approved this research or granted a formal waiver of ethical approval<br>• Include an approval number if one was obtained<br>• If the study involved *non-human primates*, add *additional details* about animal welfare and steps taken to ameliorate suffering<br>• If anesthesia, euthanasia, or any kind of animal sacrifice is part of the study, include briefly which substances and/or methods were applied<br><br>**Field Research**<br><br>Include the following details if this study involves the collection of plant, animal, or other materials from a natural setting:<br>• Field permit number<br>• Name of the institution or relevant body that granted permission	
**Data Availability**<br><br>Authors are required to make all data underlying the findings described fully available, without restriction, and from the time of publication. PLOS allows rare exceptions to address legal and ethical concerns. See the PLOS Data Policy and FAQ for detailed information.	No - some restrictions will apply

A Data Availability Statement describing where the data can be found is required at submission. Your answers to this question constitute the Data Availability Statement and **will be published in the article**, if accepted.<br><br>**Important:** Stating 'data available on request from the author' is not sufficient. If your data are only available upon request, select 'No' for the first question and explain your exceptional situation in the text box.<br><br>Do the authors confirm that all data underlying the findings described in their manuscript are fully available without restriction?	
**Describe where the data may be found in full sentences. If you are copying our sample text, replace any instances of XXX with the appropriate details.**<br><br>• If the data are **held or will be held in a public repository**, include URLs, accession numbers or DOIs. If this information will only be available after acceptance, indicate this by ticking the box below. For example: *All XXX files are available from the XXX database (accession number(s) XXX, XXX.)*.<br>• If the data are all contained **within the manuscript and/or Supporting Information files**, enter the following: *All relevant data are within the manuscript and its Supporting Information files.*<br>• If neither of these applies but you are able to provide **details of access elsewhere**, with or without limitations, please do so. For example:<br><br>*Data cannot be shared publicly because of [XXX]. Data are available from the XXX Institutional Data Access / Ethics Committee (contact via XXX) for researchers who meet the criteria for access to confidential data.*<br><br>*The data underlying the results presented in the study are available from (include the name of the third party*	The datasets used and/or analyzed during the current study are available from the corresponding author upon reasonable request.

and contact information or URL).<br>• This text is appropriate if the data are owned by a third party and authors do not have permission to share the data.<br><br>* typeset	
Additional data availability information:	

Date: 28 May 2024

Dear Editor-in-Chief,

We are delighted to submit our manuscript "Costs of outpatient services at selected primary healthcare centers in rural Bangladesh: a cross-sectional study" for consideration for publication in the PLOS ONE. We hope that this research will make a significant contribution to the existing body of knowledge since studies there is a notable gap remaining on costs of illness from both provider and user perspectives for primary outpatient in Bangladesh, hindering informed policy decisions.

This manuscript estimated the overall provider and user costs for primary outpatient care services at selected public primary healthcare facilities in Bangladesh. The findings of this study shed light on the significant gaps between prescribed and provided medicines at primary healthcare facilities, leading to higher out-of-pocket expenditure and potential adverse effects including medication non-adherence, antimicrobial resistance, and poor health outcomes. The findings will aid in formulating an evidence-based, demand-driven resource allocation strategy within healthcare financing, facilitating the goal of achieving universal health coverage. This research is relevant for academicians, healthcare professionals, and policymakers as it addresses the gaps in the cost of primary healthcare services for rural populations.

We kindly request that you consider our manuscript for publication in the PLOS ONE. This manuscript has not been submitted elsewhere, and we can assure you that the author has no conflicts of interest.

Thank you for considering our submission to your journal.

Sincerely

Md. Zahid Hasan
Assistant Scientist
Health Economics and Financing
Health Systems and Population Studies Division
International Centre for Diarrhoeal Disease Research, Bangladesh (icddr,b)

Manuscript

Click here to access/download;Manuscript;Manuscript_Final.docx

Title. Costs of outpatient services at selected primary healthcare centers in rural Bangladesh: a cross-sectional study

Short title. Costs of outpatient services at primary healthcare centers in Bangladesh

Authors

Md. Zahid Hasan[1,2,3*]

Email: md.zahid@icddrb.org

Gazi Goalm Mehdi[1]

Email: gazigolammehdi@gmail.com

Khadija Islam Tisha[1]

Email: Khadija.tisha@icddrb.org

Md Golam Rabbani[1]

Email: G.Rabbani@icddrb.org

Mohammad Wahid Ahmed[1]

Email: wahid.ahmed@icddrb.org

Dr. Subrata Paul[4]

Email: subrata.mohfw@gmail.com

Dr. Ziaul Islam[1]

Email: zia@icddrb.org

Shehrin Shaila Mahmood[1]

Email: Shaila@icddrb.org

Affiliations

1) Health Systems and Population Studies Division, icddr,b, Dhaka, Bangladesh

2) Nuffield Centre for International Health, Leeds Institute of Health Sciences, University of Leeds, Leeds, UK

3) Academic Unit of Health Economics, Leeds Institute of Health Sciences, University of Leeds, UK

[4] Health Economics Unit, Health Services Division, Ministry of Health and Family Welfare, Dhaka, Bangladesh

***Corresponding author**

Md Zahid Hasan, MSS

Postal address:

Health Systems and Population Studies Division, icddr,b,

68 Shahid Tajuddin Ahmed Sharani, Mohakhali, Dhaka-1212, Bangladesh

Cell: +8801673163613, Email address: md.zahid@icddrb.org

Abstract

Background: Upazila Health Complexes (UzHC) serve as the backbone of primary healthcare at sub-district level in rural Bangladesh, delivering comprehensive healthcare services to the grassroots level. However, not all the prescribed medicines and diagnostics services are always available at these facilities for outpatient care. This results in out-of-pocket expenditure (OOPE) to the patients for getting the prescribed medicines and diagnostics services which is not properly explored. Thus, we aimed to estimate the overall provider and user costs for outpatient care services at selected UzHCs in Bangladesh.

Methods: An ingredient-based costing approach was applied to estimate the costs for the most commonly reported illnesses at outpatient from societal perspective. A health facility survey at four purposively selected UzHCs to estimate provider costs and a patient exit survey among 452 patients of selected illness to estimate the user costs. We identified commonly reported illnesses in consultation with healthcare providers of these facilities. The difference between costs for prescribed and provided medicines at UzHCs were estimated using the market prices. Data was collected between February to March 2021.

Results: The societal cost of the common outpatient illness or symptoms varied significantly, ranging from BDT 642 to BDT 1,384 per episode. Antenatal care topped the list with the highest cost burden at BDT 1,384, followed by BDT 783 for Respiratory illness and BDT 670 for Urinary Tract Infections. On average the provider spent BDT 289 for treating an outpatient, while a patient incurred BDT 446 as OOPE. On an average a patient were expected to spent BDT 341 for purchasing medicines those were not provided from UzHCs.

Conclusion: Our study found significant gaps between prescribed and provided medicines at UzHCs, leading to higher OOPE for patients. This might result into non-compliance to prescriptions for the low-income patients increasing risks of poor health outcomes. The current healthcare resource allocation strategy does not consider the outpatient load and healthcare demand, which further exacerbates this gap. Addressing this gap requires a fundamental shift towards a demand-driven resource allocation

model in the healthcare financing strategy to ensure better treatment compliance and achieve health for all.

Keywords: Primary healthcare, Outpatient services, Out-of-pocket expenditure, Societal cost, Bangladesh.

Main text

Background

Strengthening the primary healthcare (PHC) system is vital for achieving Universal Health Coverage (UHC) as it serves as the cornerstone for delivering essential health services, promoting preventive care, and addressing health disparities at the community level [1]. Significant gaps exist in the PHC system across the majority of low-income and middle-income countries (LMICs), attributed to factors such as epidemiological transition, disease outbreaks, and inadequate governance structures [2]. Bangladesh has a multi-tiered healthcare delivery system in the public sector encompassing different levels of healthcare for the citizens across the country i.e., primary, secondary, and tertiary/specialized care [3]. The government of Bangladesh is the key actor in providing healthcare services in this pluralistic health system. At the public primary care level; there are 424 Upazila Health Complexes (UzHCs), 87 Union Health and Family Welfare Centers, 1,312 Union Sub-Centers, and 13,948 Community Clinics [4]. UzHCs offer comprehensive services, including outpatient, inpatient, and emergency care, unlike other PHC options like Union Health and Family Welfare Centers and Community Clinics, which focus solely on outpatient care. The UzHCs also serve as referral centers for the grassroots level healthcare facilities (e.g., community clinics) [5]. All these healthcare facilities are largely financed by government funds with nominal user/registration fees at UzHCs and above levels [6]. In public healthcare facilities, consultation and essential medicines are typically provided free of charge.

Bangladesh allocates only 2.88% of its gross domestic product (GDP) to the health sector, with government expenditure relative to GDP at 0.66%, placing the country among those with the lowest health spending in the Southeast Asian Region [7]. As per National Health Policy 2011, the availability of essential drugs and basic medical services in all Upazilas are prioritized. However, resources are not allocated accordingly [8]. Despite UzHCs offering both inpatient and outpatient services, the current resource allocation mechanism fails to consider the outpatient load [9]. Allocation of resources to UzHCs has primarily been determined by the number of available beds in these facilities. This creates a notable disparity in per-capita allocation with the differences in population-bed ratio. Furthermore, these variations do not seem to be justified by differences in healthcare needs across the respective areas [9]. Despite undeniable progress in healthcare access and outcomes in Bangladesh over the past two decades, evidenced by falling mortality rates and high immunization coverage, affordability remains a critical hurdle [10,11]. According to Bangladesh National Health Accounts, the OOPE for healthcare is about 68.5% which is the highest among its neighboring countries [12]. Such rising healthcare costs threaten to exclude vulnerable communities from accessing healthcare and perpetuating inequities [13,14]. The gap is further exacerbated by the limited resources available at UzHCs. Due to shortages of medicine supply and inadequate diagnostic facilities, UzHCs often cannot provide the complete dosage of medicine or diagnostic tests as prescribed. This lack of provision, compounded by high OOPE, can lead to non-compliance with healthcare providers' advice, with lasting repercussions such as disease progression, reduced functional abilities, lower quality of life, and increased strain on healthcare resources through more hospital visits and admissions.

The majority (90%) of the patients avail outpatient services compared to in-patient care at government health facilities [15]. In this context of greater demand and utilization of outpatient care at public healthcare facilities, issues of hospital cost containment, and plan to introduce

better and comprehensive outpatient services at government facilities – the policy planners need to know the cost incurring upon the government per outpatient visit and also patient's OOPE for receiving outpatient care. Detailed cost of illness studies will contribute significantly to the comprehension of the differentiating cost burden for treatment of outpatient care [16]. Few studies estimated the cost of outpatient services in public hospitals in Bangladesh [17,18]. Notably, there is a dearth of studies focusing on the outpatient service costs at UzHCs, which serve as the primary healthcare providers in rural Bangladesh. Therefore, the current study aimed to estimate the overall provider and user costs for primary outpatient service at selected UzHCs in Bangladesh.

Method

Study design

This was a cross-sectional study supplemented by an ingredient-based costing approach, conducted in 2021. The quantitative method included a health facility survey to estimate provider costs and a patient exit survey to measure the costs incurred by patients for outpatient services.

Study site

Initially, Chandpur district was purposively selected. UzHCs within the chosen district were categorized into two groups based on the number of beds, and two UzHCs from each group were further selected based on their outpatient utilization rates—one with high utilization rates and one with low utilization rates. The outpatient utilization data for these UzHCs were extracted from the local health bulletin for the past three years (2018-2020) [19]. Surveys were conducted at two 50-bedded UzHCs, namely Hajiganj UzHC and Kachua UzHC, and two 31-bedded UzHCs, namely Faridganj UzHC and Matlab North UzHC.

Sample size and data collection

A total of 452 patients were interviewed across the four selected UzHCs during the four weeks data collection period. Data were collected from exit patients who visited the healthcare facilities for pre-selected commonly reported symptoms/diseases. The most frequent outpatient diseases or symptoms were listed by consulting with the Upazila Health and Family Planning Officer and medical officers of the study UzHCs. Two data collection teams were deployed to collect data simultaneously across the four selected UzHCs. Each of the team had three trained data collectors and they spent two weeks at each UzHC for interviewing the outpatient. The patients were identified by the physicians at the selected facilities based on clinical symptoms or by the confirmation of lab tests; whichever was available. The patients with the listed disease were finally selected for interview. The number of patients interviewed varied across the UzHCs, depending on the size of the hospital and patient flow during the visit of data collectors. Pretested semi-structured questionnaires were administered by the interviewers during the face-to-face survey with the patients/caregivers and health facility staff. An electronic device-based data collection system called KoboToolbox was used for data collection. Data was collected through the following two surveys: a) Health facility survey and b) Patient exit survey conducted between February 26,2021, and March 15, 2021.

Health facility survey

To estimate the outpatient service costs from the provider's perspective, a health facility inventory survey was conducted with the facility managers, medical staff, accountants, and head assistants. Information was collected through interviews and review of relevant documents, such as the facility register for outpatient service-related statistics, to supplement and complement the survey data, along with financial documents (i.e., budget, expenditure attributable to outpatient) to estimate the total funds received and spent on outpatient services. The inventory survey also collected data on available treatments, costs of medicines, supplies, equipment used for outpatient services, personnel involved, capital items, ingredients used for diagnostic tests, number of outpatients treated, and total number of patients treated. A semi-structured questionnaire was used to conduct the facility survey.

Patient exit survey

We conducted a patient exit survey to estimate the user costs for receiving outpatient care for selected most frequent diseases or symptoms. The patient cost information was collected by interviewing the patients or accompanying persons during the exit. In the case of a patient less than 18 years old or unable to give an interview due to illness, we approached an adult caregiver of the respective patient. In this survey, questions were asked on patients' and respondents' demographic characteristics, patients' healthcare utilization, OOPE for treatment at the designated UzHCs and from any other sources within 7 days for the same cause or diseases, time devoted to care, and household assets. Additionally, a photograph of the advice slip/prescription and drugs/supplies/test report has been taken to keep evidence of treatment received on the day of the interview with the participant's consent.

Measuring the cost of illness

Generally, the total cost of an illness includes direct, indirect, and intangible costs [20]. Direct costs refer to the financial expenses incurred by health service providers and users for human resources, equipment, medicines, diagnostic tests, transportation, etc. Indirect costs represent the monetary value of productive time losses to the patient and caregivers due to the illness [20]. Intangible costs are associated with the suffering and grief from illness, which are typically challenging to measure due to their subjective nature [20]. However, in this study, we did not consider the intangible costs of illness.

Direct costs

Direct costs of illness included both direct medical costs (e.g., consultation, registration or ticket fee, medicine, diagnostic tests, etc.) and direct non-medical costs (e.g., transportation, food, informal payments, and any other types of relevant expenditure). Since all prescribed medicines were not provided to outpatients from UzHCs' pharmacy, the cost for medicines that were not provided or not available was estimated separately as expected medicine expenditure. In this regard, we collected the names and quantities for both the prescribed and provided medicine at UzHCs and estimated the costs using market price. Finally, the OOPE of the different components was estimated and separated into

medical and non-medical expenditures for all outpatients interviewed. Then average of the total medical and non-medical OOPE was estimated per outpatient for different illnesses.

On the other hand, we estimated the average treatment costs for outpatient from the provider's perspective considering the resources utilized to provide the outpatient services. This is because both outpatient and inpatient healthcare services are provided at the UzHCs. Thus, the provider costs included costs of space, furniture, equipment e.g., diagnosis and laboratory test equipment, medicine costs, staff salary, utility cost, institutional costs, and other associated costs required to provide outpatient services. To estimate the actual cost from the provider's perspective, the costs of shared capital items, staff salary, and equipment for inpatient and outpatient care were apportioned considering the proportion used for the outpatients.

Indirect costs

We utilized the human capital approach or friction cost method to estimate the value of potential productivity losses (or income loss as a proxy) as a consequence of illness [18,21,22]. The indirect costs of illness were estimated considering the income or productivity loss of patients and their caregivers for both paid and unpaid work (caregiving, household activities). The income loss from foregone non-market activities (unpaid work) was measured using occupation-specific minimum wages from the labor force survey data [23]. Thus, the patient and caregiver's income lost due to illness-related absences from work was converted into monetary value considering. In this study, we considered the individual perspective in estimating the indirect cost of illness.

Societal costs

We estimated the disease-specific societal cost of outpatient by summing up the total cost from the provider and user perspective.

Data analysis

We conducted a descriptive analysis. The characteristics of the study participants and the most frequent outpatient diseases were presented in terms of frequency (n) and percentages (%). Both component-specific and average costs of illness from the provider and user perspective were estimated and presented in Bangladeshi Taka (BDT). Average costs per patient for outpatient services were estimated in two different scenarios i) considering the drugs as prescribed which is shown as expected medicine costs and ii) the actual number of drugs provided by the facility which is shown as estimated medicine costs. Expected OOPE for medicine was defined as the costs to be incurred by patients if medicines were not provided by the UzHC and had to be purchased externally. For the unit price of medicine, the market price (medicines of local pharmaceutical companies) was considered. Data were analyzed using Stata/ SE 15.0 and Excel.

Ethics approval

Ethical approval for the study was obtained from the institutional review board of the International Centre for Diarrhoeal Disease Research, Bangladesh (icddr,b) before data collection (PR#20144). Informed written consent was taken from all the respondents prior to the interviews, and confidentiality and anonymity of their provided information were ensured. All data were collected anonymously and handled confidentially, aligning with the declaration of Helsinki.

Results

Table 1 presents the socioeconomic and demographic characteristics of the study participants. About 17% of patients were less than 5 years old, whereas about 11% were 60 and above. The proportion of female patients (66.8%) was higher compared to the male patients (33.2%). One in four patients had no institutional education. Among the patients, a sizable percentage was housewives (45.6%). The majority of the patients were married (61.5%) and around 44% of the patient's household had between three to four family members, followed by five to six members (38.7%). About 43% of patients were interviewed from Haziganj UHC followed by around 21% were from Matlab (North) UHC and the rest of 19% and 18% were from Faridganj and Kachua UHC, respectively.

Table 1. Characteristics of outpatient service users

Variables	n=452	Percentage (%)
**Age**		
Less than 5 years old	77	17.0
5-14	42	9.3
15-29	108	23.9
30-44	102	22.6
45-59	74	16.4
60 years and above	49	10.8
**Sex**		
Male	150	33.2
Female	302	66.8
**Education level**		
No institutional education	113	25.0
Up to primary	131	29.0
Secondary	157	34.7
Higher secondary and above	51	11.3
**Occupation**		
Laborer/Worker	27	6.0
Business	36	8.0
Retired/Unemployed	38	8.4
Student	51	11.3
Children	88	19.5
Housewife	206	45.6
Other (maid/self-employed)	6	1.3
**Marital status**		
Married	278	61.5
Unmarried	144	31.9
Widow/ Widower	27	6.0
Divorced/Separated	3	0.7
**Family size**		
Up to 2 members	30	6.6
3-4 members	198	43.8
5-6 members	175	38.7
7 or more members	49	10.8

**Asset Quintile**		
Lowest quintile	91	20.1
2nd quintile	90	19.9
3rd quintile	91	20.1
4th quintile	90	19.9
Highest quintile	90	19.9
**Study sites**		
Haziganj UHC	193	42.7
Kachua UHC	81	17.9
Faridganj UHC	85	18.8
Matlab (North) UHC	93	20.6

Figure 1 illustrates the most common diseases/symptoms at outpatient of the study UzHCs. The most reported diseases/symptoms among the patients included the common cold (10.0%), followed by common fever (8.0%), and back or chest pain (7.3%). Other notable ailments included Respiratory illness or breathing issues (7.1%), Gastrointestinal problems (5.1%), and Cough (4.4%). The proportion of maternity and gynaecological problems such as Menstrual problems (5.1%), Antenatal Care (ANC) (4.2%), Urinary Tract Infection (UTI) (3.8%), and Leukorrhea (2.4%) were also noticeable. Chronic conditions such as Diabetes were 6.9% of the respondents, while High Blood Pressure was 5.5%. Chronic kidney disease was less frequent, accounting for 0.7% of the respondents.

(Figure 1 will be inserted here)

Figure 1. Distribution of patients by most frequent diseases/symptoms

Table 2 shows the OOPE for outpatient healthcare utilization from the UzHCs. The estimated OOPE per outpatient was BDT 75.9 (95% CI: 66.8-85.0) at UzHCs which included both direct medical and non-medical expenditures. Among the direct medical items, the expenditure for registration and diagnostic tests were BDT 4.7 (95% CI: 4.7-4.8) and BDT 219.1 (95% CI: 93.5-344.6), respectively. Among the non-medical items, the transport expenditure was the major driver (BDT 70.4; 95% CI: 63.9-76.9), followed by expenditure for attendants (BDT 56.7: 95% CI: 6.2-107.1) and food (BDT 36.3: 95% CI: 26.3-46.4). While considering the expected OOPE for medicines and diagnostics to be

purchased/get from private sources, the expected medicine expenditure per outpatient was BDT 344.0 (95% CI: 307.7-380.3) and diagnostic expenditure was BDT 586.9 (95% CI: 428.6-745.3). The expected diagnostic expenditure was almost three times higher than the average OOPE incurred for diagnostic tests at UzHCs (Prescribed tests that were not available at UzHCs). Considering both expected OOPE for medicine and diagnostics tests, the average OOPE per outpatient was estimated to be BDT 427.3 (95% CI: 376.1-478.4) which was more than five times higher than the incurred OOPE at UzHCs. On average, a patient incurred BDT 446 as total OOPE, considering both medical and non-medical expenses, as well as expected costs for medications and diagnostic tests.

Table 2. OOPE of respondents for healthcare utilization at outpatient of the UzHCs including expected expenditure for medicine and diagnostics

OOPE components	n (%)	Mean BDT (95% CI)
**Medical expenditure items**		
Ticket/Registration fee	274 (60.6)	4.7 (4.7-4.8)
Diagnostic expenditure incurred at UzHCs	11 (2.4)	219.1 (93.5-344.6)
Medical expenditure	**452 (100.0)**	**8.2 (4.1-12.3)**
**Non-medical expenditure items**		
Food expenditure	60 (13.3)	36.3 (26.3-46.4)
Attendant expenditure	12 (2.7)	56.7 (6.2-107.1)
Transport expenditure	384 (85.0)	70.4 (63.9-76.9)
Other expenditure (e.g., tips)	11 (2.4)	65.0 (13.2-116.8)
Non-medical expenditure	**452 (100.0)**	**67.7 (60.2-75.2)**
**Subtotal [a)]**	**452 (100.0)**	**75.9 (66.8-85.0)**
**Expected OOPE for medicine and diagnostics**		
Medicine	385 (98.5)	344 (307.7-380.3)
Diagnostic	59 (15.1)	586.9 (428.6-745.3)
Subtotal	**391 (100.0)**	**427.3 (376.1-478.4)**
**Total OOPE [b)]**	**452 (100.0)**	**445.5(397.3-493.7)**
USD to BDT conversion rate, 1USD= 85.0 BDT		

[a)] Without OOPE for expected medicine and diagnostics
[b)] Including expected OOPE for medicines and diagnostics

Table 3 shows the average costs per outpatient from the provider's perspective. Capital costs per outpatient service were estimated to be BDT 10.5 of which space cost was BDT 9.4. On the other hand, staff salary was identified as a major cost driver for recurrent items with BDT 262.5, followed by others and utilities estimated at BDT 9.8 and BDT 3.8, respectively. However, combined with the total capital and recurrent items, the estimated total cost of the provider was estimated at BDT 289.0 for an episode of outpatient service.

Table 3. Average costs per outpatient case from the provider perspective (n=4)

Cost components	Cost items	Mean BDT
**Capital items**	Space	**9.4**
	Furniture	**0.3**
	Equipment	**0.9**
Sub-total of capital		**10.5**
**Recurrent items**	Staff salary	**262.5**
	Cleaning	**2.9**
	Utilities	**3.8**
	Others	**9.3**
**Subtotal of recurrent**		**278.5**
**Total provider cost per patient**		**289**
USD to BDT conversion rate, 1 USD= 85.0 BDT		

Table 4 shows the expected medicine cost across the symptoms/diseases. Expected OOPE for medicine (costs for required medicines to be purchased from outside) was the highest for ANC at BDT 594.6, followed by UTI at BDT 497.2 and for Menstrual problems at BDT 474.4. The expected cost was found to be the lowest for children’s ear problems at BDT 137.5. The overall expected medicine cost was estimated to be BDT 341.3 per patient.

Table 4. Expected cost of medicine and gap between the costs of prescribed and provided medicine

Diseases/ Symptoms	n (%)	Expected Medicine costs as per prescription	* Estimated Medicine costs as per Provision	Expected Medicine costs (gap of costs between required as per prescription and provided medicine)
Common cold	31 (8.3)	280.6 (167.8-393.5)	115.3 (28.9-201.6)	165.4 (81.8-249.0)
Common fever	26 (6.9)	219.6 (137.4-301.8)	54.6 (27.6-81.6)	165.0 (88.1-241.9)
Cough	16 (4.3)	287.3 (177.7-396.9)	121.6 (56.9-186.2)	165.8 (92.9-238.6)
Back or chest pain	28 (7.5)	449.3 (320.4-578.3)	58.6 (39.0-78.3)	390.7 (259.7-521.7)
Gastrointestinal problems	21 (5.6)	302.5 (197.4-407.6)	61.8 (40.3-83.3)	240.7 (132.7-348.7)
Respiratory illness	32 (8.5)	572.5 (402.1-742.8)	179.3 (110.0-248.5)	393.2 (259.5-527.0)
Urinary Tract infection	17 (4.5)	666.9 (415.3-918.6)	169.7 (79.9-259.5)	497.2 (268.0-726.4)
Skin disease	19 (5.1)	354.3 (143.3-565.3)	73.3 (25.5-121.0)	281.1 (89.2-472.9)
Diarrhea	14 (3.7)	223.3 (109.8-336.8)	81.9 (36.5-127.3)	141.4 (27.4-255.4)
Lower abdominal pain	13 (3.5)	356.6 (214.1-499.2)	124.9 (9.5-240.3)	231.7 (108.9-354.4)
Pneumonia	10 (2.7)	584.5 (400.4-768.6)	143.4 (-12.1-298.9)	441.1 (251.3-630.9)
Children's ear problems	8 (2.1)	194.9 (127.1-262.7)	57.4 (3.4-111.4)	137.5 (68.9-206.1)
Diabetics	30 (8.0)	612.9 (443.2-782.6)	284.3 (188.3-380.3)	328.6 (192.0-465.2)
High blood pressure	23 (6.1)	584.0 (349.0-818.9)	124.0 (63.1-184.9)	460.0 (237.3-682.6)
Antenatal care	19 (5.1)	727.2 (517.4-937.0)	132.6 (49.8-215.5)	594.6 (361.2-827.9)
Menstrual problem	21 (5.6)	535.0 (333.8-736.3)	60.7 (21.9-99.5)	474.4 (297.8-651.0)
Weakness	27 (7.2)	387.6 (259.0-516.2)	82.9 (56.6-109.2)	304.7 (171.4-438.1)
Malnutrition	9 (2.4)	226.2 (49.4-403.1)	16.7 (4.5-28.8)	209.6 (40.7-378.4)
Leukorrhea	11 (2.9)	452.7 (286.6-618.9)	155.1 (41.6-268.5)	297.6 (123.5-471.8)
**Total**	**375 (100.0)**	**458.2 (395.7-520.7)**	**116.9 (99.5-134.2)**	**341.3 (282.8-399.8)**

Table 5 shows the societal cost of the most common outpatient disease or symptoms in UzHCs. Among the diseases, the societal costs for ANC were the highest (BDT 2019.8; 95% CI: 1554.1-2485.5), followed by costs for Diabetes (BDT 1211: 95% CI: 957.9-1464.2) and Cough (BDT 1203.9: 95% CI: 915.6-1492.2).

Table 5. Societal cost for most frequent outpatient diseases or symptoms

Most frequent outpatient diseases/symptoms	Patients OOPE	UzHCs Medicine costs	Provider costs (except medicine)	Indirect cost	Societal costs
	Mean (95% CI)	Mean (95% CI)	Mean (95% CI)	Mean (95% CI)	Mean (95% CI)
Common cold	228.8 (141 - 316.7)	115.3 (28.9 - 201.6)	271.1 (248.2 - 294.1)	65.9 (37.4 - 94.5)	681.1 (560.3 - 802.0)
Common fever	429.6 (195.9 - 663.2)	54.6 (27.6 - 81.6)	262.8 (230.6 - 295)	65.6 (36.1 - 95.1)	812.6 (586.4 - 1038.8)
Cough	281.5 (178.5 - 384.5)	121.6 (56.9 - 186.2)	249.5 (213.2 - 285.8)	120.4 (54.7 - 186.2)	772.9 (576.2 - 969.6)
Back or chest pain	487.4 (329.8 - 644.9)	58.6 (39 - 78.3)	281 (251.3 - 310.8)	83.1 (60.6 - 105.7)	910.2 (754.6 - 1065.7)
Gastrointestinal problems	336.3 (233.2 - 439.5)	61.8 (40.3 - 83.3)	272 (241.9 - 302.1)	113.0 (49.7 - 176.3)	783.1 (635.4 - 930.8)
Respiratory illness	785.2 (243.9 - 1326.4)	179.3 (110 - 248.5)	276.9 (255.6 - 298.3)	67.7 (41.3 - 94.1)	1059.8 (865.4 - 1254.2)
Cough	671.5 (426.6 - 916.4)	169.7 (79.9 - 259.5)	260.1 (216.5 - 303.6)	102.6 (55.2 - 149.9)	1203.9 (915.6 - 1492.2)
Skin disease	378.8 (157.2 - 600.5)	73.3 (25.5 - 121)	262.9 (228.1 - 297.7)	126.1 (21.4 - 230.7)	841.1 (491.2 - 1190.9)
Diarrhea	205.3 (73.2 - 337.4)	81.9 (36.5 - 127.3)	264.6 (226.7 - 302.4)	90.3 (39.9 - 140.8)	642.1 (477.1 - 807.1)
Lower abdominal pain	291.2 (139.6 - 442.7)	124.9 (9.5 - 240.3)	272.8 (226.8 - 318.7)	74.3 (43.5 - 105.0)	763.1 (602.2 - 924.1)
Pneumonia	493.1 (284.2 - 702)	143.4 (-12.1 - 298.9)	283.5 (239.8 - 327.2)	88.8 (40.2 - 137.4)	1008.8 (816.5 - 1201.1)
Children's ear problems	227.3 (125.8 - 328.7)	57.4 (3.4 - 111.4)	323.4 (289.3 - 357.6)	88.2 (48.9 - 127.4)	696.2 (526.1 - 866.3)
Diabetes	542.7 (330.6 - 754.8)	284.3 (188.3 - 380.3)	288.2 (260.6 - 315.8)	95.8 (63.6 - 128.0)	1211.0 (957.9 - 1464.2)
High blood pressure	568.9 (336 - 801.7)	124 (63.1 - 184.9)	285.7 (257.8 - 313.6)	85.9 (52.3 - 119.5)	1064.5 (809.5 - 1319.4)
Antenatal care	1383.8 (931.8 - 1835.8)	132.6 (49.8 - 215.5)	286.5 (242.5 - 330.4)	216.9 (84.6 - 349.3)	2019.8 (1554.1 - 2485.5)
Menstrual problem	705.1 (461.4 - 948.7)	60.7 (21.9 - 99.5)	290.8 (262.5 - 319)	82.8 (61.2 - 104.3)	1139.3 (882.1 - 1396.6)
Weakness	381.1 (247.4 - 514.9)	82.9 (56.6 - 109.2)	277.8 (251 - 304.6)	89.7 (59.0 - 120.4)	831.5 (696.3 - 966.73)
Malnutrition	284.6 (107.6 - 461.5)	16.7 (4.5 - 28.8)	300.6 (-)	163.7 (54.4 - 273.1)	765.5 (493.5 - 1037.6)
Leukorrhea	604.1 (62.6 - 1145.6)	155.1 (41.6 - 268.5)	312.2 (270.7 - 353.6)	74.1 (52.4 - 95.8)	1145.5 (613.8 - 1677.1)

Discussion

This study aimed to estimate the societal cost of outpatient healthcare services for common diseases/ symptoms in selected UzHCs of rural Bangladesh. The study highlighted an often-overlooked aspect of OOPE incurred by patients while utilizing PHC at public hospitals. The findings revealed that there is a gap between the cost of prescribed and provided medicines at UzHCs ranging between BDT 137 (USD 1.6) to BDT 595 (USD 7.0) per outpatient user. This is because, although prescribed medicines at public hospitals are meant to be "free", often they are not provided due to unavailability of stock or rules of medicine disbursement. Thus, patients are required to purchase the prescribed medicines from private pharmacies and get diagnostics tests at private diagnostic centers. Though the Constitution of Bangladesh made an obligation for the Government of Bangladesh to ensure good health for all its citizens, the study revealed that patients incurred a significant amount of expenditure to utilize primary care at the outpatient. This finding is aligned with the result of a national-level study that estimated that 68.5 % of OOPE is incurred by households directly to providers in accessing healthcare goods and services [23]. Though essential medicines and diagnostic services were provided by public health facilities, a sizable number of patients (about 85%) still needed to purchase medicine and diagnostic services. It was estimated that the diagnostic services cost is the main driver of the expected OOPE followed by medicine costs which are justified by a study conducted by MoHFW [23].

We found that the societal costs for antenatal care (BDT 2020) were the highest among the included diseases followed by Diabetes (BDT 1211) and Cough (BDT 1204). Several studies estimated the ANC cost which is comparatively lower than our estimates [24–26]. There might be several reasons for this difference. For example, we have considered the expected OOPE if the patients purchase the complete prescribed medicines and diagnostic services and all cost items from the provider side; whereas the other studies either considered only incurred OOPE or did not include infrastructure, capital equipment, overhead or other costs in calculating service provision costs [24].

The findings indicated that the patients had to pay a significant amount of OOPE for PHC in outpatient despite the existence of free public health services. The average OOPE for outpatient healthcare incurred at the UzHCs was estimated at BDT 75.9 (USD 0.89). On the other hand, when we estimated the average OOPE considering the expected medicine and diagnostic expenditure (as per the

prescription), the overall OOPE increased by five times (BDT 75.9 vs BDT 427.3). Studies in rural Bangladesh [27–29] and other LMICs [30–32] consistently identified medicine and diagnostic costs as the key drivers of OOPE. In LMICs, OOPE is around 35.25% of the total healthcare expenditure which is a significant barrier to accessing and utilizing healthcare services [33]. From the provider's perspective, the average cost per outpatient case (excluding medicine cost) was estimated at BDT 289 (USD 3.4), where recurrent cost (96%) was the major cost driver. This finding is similar to a study conducted in Ghana, where the estimated cost per outpatient attendance was estimated at USD 4.51 at district-level hospitals [34].

The lack of provisioning of a full course of free prescribed medicines at UzHCs increases the burden of OOPE for healthcare and increases the tendency of non-compliance among care seekers. While skipping a few doses might seem harmless in hindsight, it can have serious long-term consequences. Stopping medication prematurely, often when symptoms fade, is a common form of non-compliance and a significant contributor to antimicrobial resistance (AMR) [35]. Studies have documented this link, highlighting the non-compliance as a major driver of AMR in Bangladesh [36–39]. Consequently, the inadequate medicine supplies at UzHCs can trigger a series of adverse events, a poor quality of life, and ultimately, a heavier burden on healthcare resources due to increased hospital visits and admissions [40–43]. On the other hand, the inadequate mix of human resources and infrastructure within UzHC's subsidized diagnostic testing services proved to be insufficient as per the prescribed diagnostic tests [44]. This shortfall compelled the patients to seek services outside the UzHCs, leading to a rise in OOPE burdens [27].

The study's findings highlighted a critical gap between medicine demand and supply within the rural PHC system, demanding immediate policy attention. Current policies, which solely rely on inpatient services to allocate resources for UzHCs, neglect the significant patient load at outpatient departments [9]. This inadequate medicine provision further exacerbates the burden of OOPE to the patients which is a major barrier to accessing healthcare services for the vulnerable communities. While the government's endeavour to achieve universal health coverage (UHC) promises universal access to quality healthcare, this gap significantly hinders healthcare utilization in rural Bangladesh. Bridging this gap necessitates policy-level changes, including strengthening the existing rural healthcare system

by guaranteeing a steady supply of medicines and increasing the number of essential diagnostic tests at UzHCs level. Additionally, curbing the burden of OOPE through optimized resource allocation and expanded health insurance coverage is crucial. A fundamental shift towards a demand-driven allocation model in the healthcare financing strategy is required. Policymakers need to prioritize resource allocation based on the population's healthcare needs, including ensuring the provision of complete prescribed medication dosages.

The study has some limitations. The costs of medicines and diagnostic tests were estimated based on market prices. Since the costs of medicines and diagnostic ingredients vary from one pharmaceutical company to another, the calculated cost may not precisely reflect the exact unit cost. However, this will give an idea of how much budget will be required for the provisioning of a full course of medicines for the considered illness. The list of the most frequent diseases was prepared in consultation with healthcare providers at UzHCs. Thus, there may be a presence of response bias. Another significant limitation to consider is related to the study design. Since this is a cross-sectional study and data were collected over a two-month period, seasonal bias may have occurred, potentially influencing the cost estimation or there might be difference in the list of common diseases considered for costing. Despite these limitations, the empirical evidence from this study holds significant strength. The findings are expected to provide valuable insights to policymakers involved in health budget allocation and strengthening the PHC system in Bangladesh. Furthermore, a large-scale, well-planned national prospective study could be undertaken to estimate more precise social costs of outpatient services at the UzHCs.

Conclusion

Despite Bangladesh's commitment to UHC, significant gaps remain in rural PHC. Inadequate medicine supply forces patients to incur OOPE for a complete course of treatment, hindering access to essential treatment and having potential side effects including medication non-adherence, antimicrobial resistance, and poor health outcomes. To bridge this gap, policies must prioritize outpatient needs, guarantee complete medication availability, and curb burdens of OOPE through optimized resource allocation and expanded health insurance coverage. A fundamental shift towards a demand-driven

financing model is crucial to strengthening the rural PHC system in increasing supplies of medicines and providing required diagnostic services which will ensure access to essential healthcare services for all patients.

Declarations

Acknowledgments

We sincerely acknowledge the funding support provided by the Health Economics Unit, Ministry of Health and Family Welfare. We also acknowledge the authority of Haziganj, Kachua, Faridganj, and Matlab North UzHC for their cooperation and support in conducting the survey. icddr,b is also thankful to the Governments of Bangladesh and Canada for providing unrestricted/ institutional support.

Authors' contributions

MZH, MWA, and ZI conceived and designed the study. MZH, GGM, MGR, and MWA performed data cleaning and analysis. MZH, KIT, MGR, and SP drafted the manuscript. ZI and SSM critically reviewed and edited the manuscript. All authors reviewed, edited, and approved the final manuscript.

Funding

The study was funded by the Health Economics Unit (HEU), Health Services Division, Ministry of Health and Family Welfare, the Government of Bangladesh (Grant #: GR-01964).

Competing interests

MZH, GGM, KIT, MGR, MWA, ZI, and SSM declare that they have no competing interests. SP is affiliated with the funding agency. However, Views expressed in this paper do not necessarily reflect the views of the HEU authority.

References

1. Sacks E, Schleiff M, Were M, Chowdhury AM, Perry HB. Communities, universal health coverage and primary health care. Bull World Health Organ. 2020;98: 773–780.

doi:10.2471/BLT.20.252445

2. Saif-Ur-Rahman KM, Mamun R, Anwar I. Identifying gaps in primary healthcare policy and governance in low-income and middle-income countries: protocol for an evidence gap map. BMJ Open. 2019;9: e024316. doi:10.1136/bmjopen-2018-024316
3. Ahmed SM, Alam BB, Anwar I, Begum T, Haque R, Khan JA, et al. Bangladesh Health System Review. Health Systems in transition. 2015. Available: https://apps.who.int/iris/bitstream/handle/10665/208214/9789290617051_eng.pdf?sequence=1&isAllowed=y
4. MIS. Health Bulletin 2020. Dhaka; 2022.
5. Riaz BK, Ali L, Ahmad SA, Islam MZ, Ahmed KR, Hossain S. Community clinics in Bangladesh: A unique example of public-private partnership. Heliyon. 2020;6: e03950. doi:10.1016/j.heliyon.2020.e03950
6. Islam, Ahsan B. Health System Financing in Bangladesh: A Situation Analysis. Am J Econ Financ Manag. 2015;1: 494–502.
7. Health Economics Unit. Public Expenditure Review 1997-2020. Heal Serv Div. 2020. doi:10.1596/35064
8. Ministry of Health and Family Welfare. National Health Policy 2011. 2012. Available: http://mohfw.gov.bd/index.php?option=com_content&view=article&id=74&Itemid=92&lang=en
9. Ensor T, Hossain A, Sen PD, Ali L, Begum SA, Moral H. Geographic resource allocation in Bangladesh. Heal Policy Res South Asia Build Capacit Reform. 1998.
10. Mitra DK, Mridha MK. Sustaining progress in the health landscape of Bangladesh. Lancet Glob Heal. 2023;11: e1838–e1839. doi:10.1016/S2214-109X(23)00494-1
11. Chowdhury AMR, Bhuiya A, Chowdhury ME, Rasheed S, Hussain Z, Chen LC. The Bangladesh paradox: exceptional health achievement despite economic poverty. Lancet. 2013;382: 1734–1745. doi:10.1016/S0140-6736(13)62148-0
12. Health Economics Unit. Bangladesh National Health Accounts 1997–2020. Dhaka; 2022.
13. Asante A, Price J, Hayen A, Jan S, Wiseman V. Equity in Health Care Financing in Low- and

Middle-Income Countries: A Systematic Review of Evidence from Studies Using Benefit and Financing Incidence Analyses. PLoS One. 2016;11: e0152866. doi:10.1371/journal.pone.0152866

14. Rahman T, Gasbarro D, Alam K. Financial risk protection from out-of-pocket health spending in low- and middle-income countries: a scoping review of the literature. Heal Res Policy Syst. 2022;20: 83. doi:10.1186/s12961-022-00886-3
15. DGHS. Health Bulletin, 2018. Management Information System (MIS), Directorate General of Health Services, Ministry of Health and Family Welfare. Dhaka, Bangladesh; 2018.
16. Rice DP. Cost of illness studies: what is good about them? Inj Prev. 2000;6: 177–179. doi:10.1136/ip.6.3.177
17. Eliya R, Kasthuri G, Alwis S De. Impact of Maternal and Child Health Private Expenditure on Poverty and Inequity in Bangladesh : Maternal and Child Health Expenditure in Bangladesh. ADB. 2012. Available: https://ideas.repec.org/p/asd/wpaper/rpt135433-3.html#abstract
18. Pavel MS, Chakrabarty S, Gow J. Cost of illness for outpatients attending public and private hospitals in Bangladesh. Int J Equity Health. 2016;15: 167. doi:10.1186/s12939-016-0458-x
19. MIS. Local Health Bulletin. In: DGHS, MOFHW [Internet]. 2021. Available: https://dashboard.dghs.gov.bd/pages/lhb/lhb_search.php?type=50
20. Drummond M, Drummond MF, McGuire A. Economic evaluation in health care: merging theory with practice. Oxford University Press; 2001.
21. Wyss D, Vehorn CL, Wagner DP, Berk A, Landefeld JS, Resnick C, et al. Cost of disease and illness in the United States in the year 2000. Public Health Rep. 1978;93: 493–588.
22. Koopmanschap MA, Rutten FF, van Ineveld BM, van Roijen L. The friction cost method for measuring indirect costs of disease. J Health Econ. 1995;14: 171–189. doi:10.1016/0167-6296(94)00044-5
23. MoHFW. Bangladesh National Health Accounts 1997-2020 (BNHA-VI). Dhaka; 2022.
24. Jo Y, Alland K, Ali H, Mehra S, Lefevre AE, Pak S, et al. Antenatal care in rural Bangladesh: Current state of costs, content and recommendations for effective service delivery. BMC Health Serv Res. 2019;19: 1–13. doi:10.1186/s12913-019-4696-7

25. Rahman M, Rob U, Noor FR, Bellows B. Out-of-pocket expenses for maternity care in rural Bangladesh: a public-private comparison. Int Q Community Health Educ. 33: 143–157. doi:10.2190/IQ.33.2.d

26. Keya KT, Rob U, Rahman M, Bajracharya A, Bellows B. Distance, transportation cost, and mode of transport in the utilization of facility-based maternity services: evidence from rural Bangladesh. Int Q Community Health Educ. 2014;35: 37–51. doi:10.2190/IQ.35.1.d

27. Ahsan SM, Hamid SA, Barua S. Utilisation of Formal Health Care and Out-of-Pocket Payments. 2012.

28. Hamid SA, Ahsan SM, Begum A. Disease-specific impoverishment impact of out-of-pocket payments for health care: Evidence from rural Bangladesh. Appl Health Econ Health Policy. 2014;12: 421–433. doi:10.1007/s40258-014-0100-2

29. Shahadat Hossain Siddiquee M. Healthcare-Seeking Behavior and Out-of-Pocket Payments in Rural Bangladesh: A Cross-Sectional Analysis. Psychol Behav Sci. 2018;7: 45. doi:10.11648/j.pbs.20180703.12

30. Teni FS, Gebresillassie BM, Birru EM, Belachew SA, Tefera YG, Wubishet BL, et al. Costs incurred by outpatients at a university hospital in northwestern Ethiopia : a cross-sectional study. BMC Health Serv Res. 2018; 1–10.

31. Laokri S, Soelaeman R, Hotchkiss DR. Assessing out-of-pocket expenditures for primary health care: how responsive is the Democratic Republic of Congo health system to providing financial risk protection? BMC Health Serv Res. 2018;18: 451. doi:10.1186/s12913-018-3211-x

32. Semwal V, Singh AK. Financial Burden of Out-of-Pocket Expenditures for Primary Health Care in Hilly Areas of Garhwal Region , Uttarakhand , North India. 2017;11: 11–14. doi:10.7860/JCDR/2017/24761.9646

33. The World Bank. Out-of-pocket expenditure (% of current health expenditure) - Low & middle income | Data. [cited 27 Dec 2022]. Available: https://data.worldbank.org/indicator/SH.XPD.OOPC.CH.ZS?locations=XO

34. Dalaba MA, Welaga P, Matsubara C. Cost of delivering health care services at primary health

facilities in Ghana. 2017; 1–11. doi:10.1186/s12913-017-2676-3

35. Ayukekbong JA, Ntemgwa M, Atabe AN. The threat of antimicrobial resistance in developing countries: causes and control strategies. Antimicrob Resist Infect Control. 2017;6: 47. doi:10.1186/s13756-017-0208-x

36. Hoque R, Ahmed SM, Naher N, Islam MA, Rousham EK, Islam BZ, et al. Tackling antimicrobial resistance in Bangladesh: A scoping review of policy and practice in human, animal and environment sectors. Klein EY, editor. PLoS One. 2020;15: e0227947. doi:10.1371/journal.pone.0227947

37. Bonna AS, Pavel SR, Ferdous J, Khan SA, Ali M. Antibiotic resistance: An increasingly threatening but neglected public health challenge in Bangladesh. Int J Surg Open. 2022;49: 100581. doi:10.1016/j.ijso.2022.100581

38. Sutradhar K. Irrational Use of Antibiotics and Antibiotic Resistance in Southern Rural Bangladesh: Perspectives from Both the Physicians and Patients. Annu Res Rev Biol. 2014;4: 1421–1430. doi:10.9734/ARRB/2014/8184

39. Ahmed I, Rabbi MB, Sultana S. Antibiotic resistance in Bangladesh: A systematic review. Int J Infect Dis. 2019;80: 54–61. doi:10.1016/j.ijid.2018.12.017

40. Kane S, Shaya F. Medication Non-adherence is Associated with Increased Medical Health Care Costs. Dig Dis Sci. 2008;53: 1020–1024. doi:10.1007/s10620-007-9968-0

41. Mongkhon P, Ashcroft DM, Scholfield CN, Kongkaew C. Hospital admissions associated with medication non-adherence: a systematic review of prospective observational studies. BMJ Qual Saf. 2018;27: 902–914. doi:10.1136/bmjqs-2017-007453

42. Chiatti C, Bustacchini S, Furneri G, Mantovani L, Cristiani M, Misuraca C, et al. The Economic Burden of Inappropriate Drug Prescribing, Lack of Adherence and Compliance, Adverse Drug Events in Older People. Drug Saf. 2012;35: 73–87. doi:10.1007/BF03319105

43. Walsh CA, Cahir C, Tecklenborg S, Byrne C, Culbertson MA, Bennett KE. The association between medication non-adherence and adverse health outcomes in ageing populations: A systematic review and meta-analysis. Br J Clin Pharmacol. 2019;85: 2464–2478. doi:10.1111/bcp.14075

44. Ahmed S, Siddique A, Iqbal A, Rahman PN, Islam MN, Sobhan MA, et al. Causes for Hospitalizations at Upazila Health Complexes in Bangladesh. J Heal Popul Nutr. 2010;28. doi:10.3329/jhpn.v28i4.6047

Figure

Click here to access/download;Figure;Figure 1.png

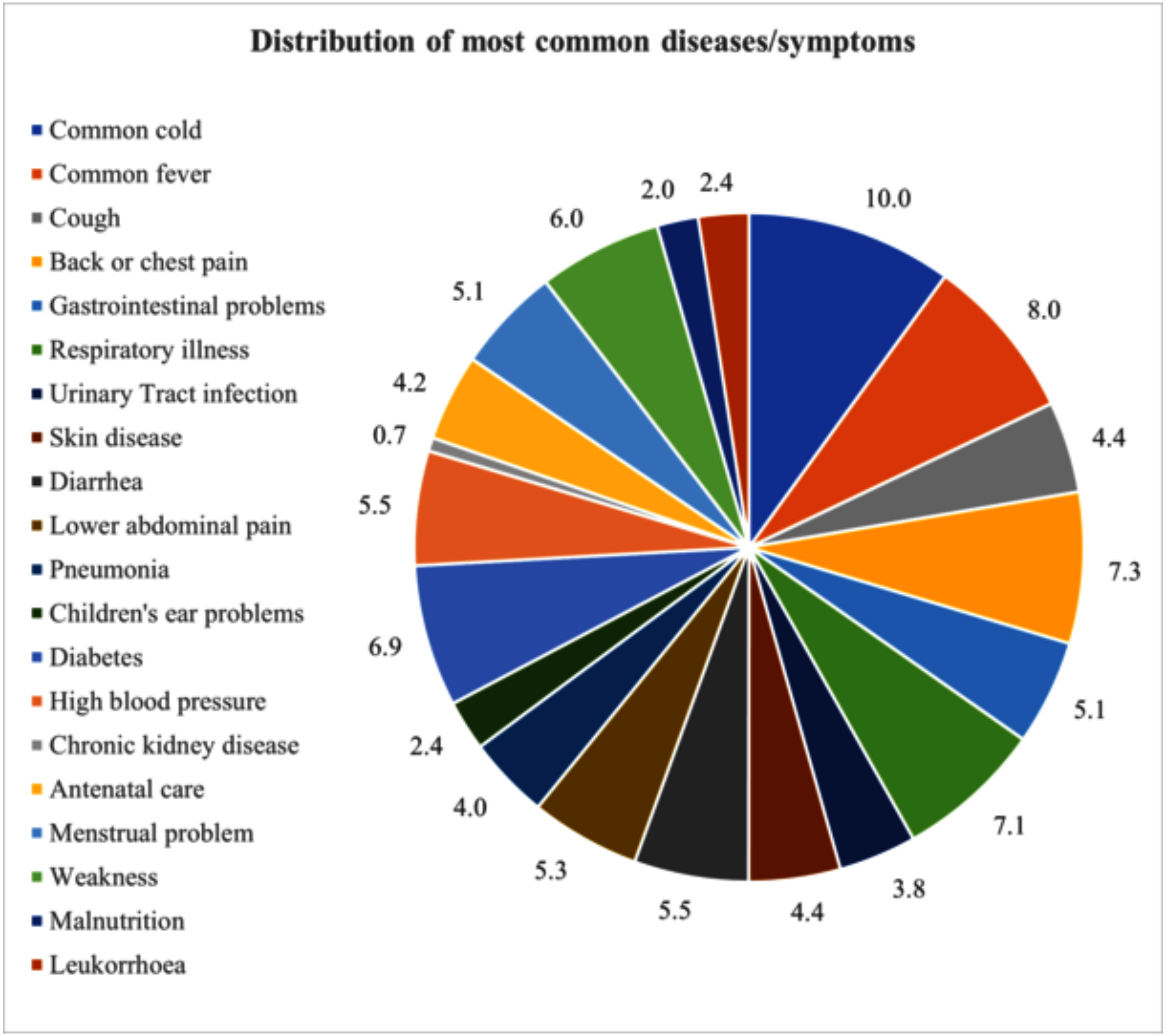

Ethical Approval

Click here to access/download
Other
Ethical approval letter.pdf
