# Supplementary material for: Costs of outpatient services at selected primary healthcare centers in Bangladesh: A cross-sectional study
Source: PLoS One. 2025 Jan 14;20(1):e0317317. doi: 10.1371/journal.pone.0317317 (PMC11731759; doi:10.1371/journal.pone.0317317)
Supplement: Attachment — Submitted filename: PLOS Review response on Cost.docx [file pone.0317317.s005.docx]

**METHODOLOGY**

**Sample size**

Kindly show how you arrived at your sample size of 452

**Sampling Technique**

Though the authors made some attempts under study site and sampling size/data collection to explain the sampling technique used by this study, the explanation is still not clear. Therefore, kindly create a section titled “Sampling Technique” and explain step by step on how you selected your facilities and respondents. It appears you used a multistage sampling technique and you stated how you purposively selected the primary health centers used, however you did not explain how you allocated questionnaires to each of the centers and the number of questionnaires allocated. You did not also clearly state how and technique used in respondents recruitment over the study period or are these patients also selected using convenience sampling by the physician (a convenience sampling (non-probabilistic technique) may introduce bias into you study and reduce its soundness scientifically).

**RESULTS**

**Table 4**

It will be beneficial if an additional column is created to show percentage of cost paid by the patients out of the “Expected Medicine costs as per prescription´ for each diseases and total cost. This will show the percentage OOPE for each diseases and also cumulatively.

E.g

| **Diseases/ Symptoms** | **n (%)** | **Expected Medicine costs as per prescription** | *** Estimated Medicine costs as per Provision** | **Expected Medicine costs (gap of costs between required as per prescription and provided medicine)** | **Percentage of difference (OOPE)** |
| --- | --- | --- | --- | --- | --- |
| Common cold | 31 (8.3) | 280.6 (167.8-393.5) | 115.3 (28.9-201.6) | 165.4 (81.8-249.0) | 58.9%  (165.4/280.6 * 100) |
|  |  |  |  |  |  |
|  |  |  |  |  |  |
|  |  |  |  |  |  |
|  |  |  |  |  |  |
| Total | 375 (100.0) | 458.2 (395.7-520.7) | 116.9 (99.5-134.2) | 341.3 (282.8-399.8) | 74.5% (341.3/458.2*100) |

You may not need to include the figures used in calculation (marked in red). It was just included here to guide on how the calculation was gotten)

**DISCUSSION**

Include the percentage of OOPE gotten in this study (as gotten in new table 4) in your discussion part from line 297 to 307. This will enable you make clearer comparison with the “national-level study” value quoted on line 306.

**CONCLUSION**

The conclusion should also reflect your study outcome based on you objectives
