# Supplementary material for: Costs of outpatient services at selected primary healthcare centers in Bangladesh: A cross-sectional study
Source: PLoS One. 2025 Jan 14;20(1):e0317317. doi: 10.1371/journal.pone.0317317 (PMC11731759; doi:10.1371/journal.pone.0317317)
Supplement: Attachment — Submitted filename: Point by point response for reviewers.docx [file pone.0317317.s007.docx]

**Response to Editor:**

**--------------------------**

**Comment 1.** Please ensure that your manuscript meets PLOS ONE's style requirements, including those for file naming. The PLOS ONE style templates can be found at

https://journals.plos.org/plosone/s/file?id=wjVg/PLOSOne_formatting_sample_main_body.pdf and <https://journals.plos.org/plosone/s/file?id=ba62/PLOSOne_formatting_sample_title_authors_affiliations.pdf>

**Response:** Thanks for sharing the links. We have now formatted the manuscript following the PLOS ONE guideline.

**Comment 2.** Please provide additional details regarding participant consent. Your study included minors, state whether you obtained consent from parents or guardians. If the need for consent was waived by the ethics committee, please include this information.

**Response:** Thank you for the comment. While the study included data for individuals below 18 years of age, minors themselves were not interviewed. Instead, for patients under 18 or those unable to participate due to illness, interviews were conducted with their parents or an adult caregiver ***(Please see page 7; lines 149-150)***. We have now revised the ethical consideration sub-section and provided additional details regarding participant consent ***(Please see page 11; lines 247-252).***

**Comment 3.** We note that the grant information you provided in the ‘Funding Information’ and ‘Financial Disclosure’ sections do not match. When you resubmit, please ensure that you provide the correct grant numbers for the awards you received for your study in the ‘Funding Information’ section.

**Response:** Many thanks for identifying the inconsistency. We have now checked and made the necessary corrections as suggested.

**Comment 4.** Thank you for stating the following in the Acknowledgments Section of your manuscript:

“We sincerely acknowledge the funding support provided by the Health Economics Unit, Ministry of Health and Family Welfare. We also acknowledge the authority of Haziganj, Kachua, Faridganj, and Matlab North UzHC for their cooperation and support in conducting the survey. icddr,b is also thankful to the Governments of Bangladesh and Canada for providing unrestricted/ institutional support.”

We note that you have provided funding information that is currently declared in your Funding Statement. However, funding information should not appear in the Acknowledgments section or other areas of your manuscript. We will only publish funding information present in the Funding Statement section of the online submission form.

Please remove any funding-related text from the manuscript and let us know how you would like to update your Funding Statement. Currently, your Funding Statement reads as follows:

“The study was funded by the Health Economics Unit (HEU), Health Services Division, Ministry of Health and Family Welfare, the Government of Bangladesh (Grant #: GR-01964).”

Please include your amended statements within your cover letter; we will change the online submission form on your behalf.

**Response:** Thanks for the instructions. We have now removed funding-related information from the manuscript and as suggested we have provided the funding information in the cover letter for your information. We appreciate you taking care of the changes to the online submission form on my behalf. ***(Please see page 21; lines 419-423)***

***Funding information***

*The study was funded by the Health Economics Unit (HEU), Health Services Division, Ministry of Health and Family Welfare, the Government of Bangladesh (Grant #: GR-01964)*

**Comment 5.** In the online submission form, you indicated that [The datasets used and/or analyzed during the current study are available from the corresponding author upon reasonable request.].

All PLOS journals now require all data underlying the findings described in their manuscript to be freely available to other researchers, either 1. In a public repository, 2. Within the manuscript itself, or 3. Uploaded as supplementary information.

This policy applies to all data except where public deposition would breach compliance with the protocol approved by your research ethics board. If your data cannot be made publicly available for ethical or legal reasons (e.g., public availability would compromise patient privacy), please explain your reasons on resubmission and your exemption request will be escalated for approval.

**Response:** Thank you for sharing PLOS journals' data sharing policy. We would like to confirm that the deidentified data used in this manuscript in included as supplementary material.

**Response to Additional Editor:**

**-----------------------------------------**

**Response:** We have addressed the additional comments under the reviewers’ comments section, as they are similar.

**Responses to Reviewer 1:**

**--------------------------------**

**Comment on Methodology**

**Comment 1:** Sample size: Kindly show how you arrived at your sample size of 452

**Response:** Many thanks for this comment. We have arrived at our sample size of 452 based on the following approach *“Since the primary focus of this study was on estimating the cost of outpatient service, a single population mean formula was used to determine the sample size. A prior study reported that the standard deviation of cost for outpatient service at public hospitals in Bangladesh was USD 124. We used this value to calculate the sample size for the UzHCs. Assuming a 95% confidence level and a 10% margin of error from the mean costs, we determined that 340 respondents would be required for the exit survey. Further, taking into account a 1.2 design effect and a 10% non-response rate, the estimated sample size increased to 512. This sample was proportionally allocated across the four UzHCs based on the previous year's utilization rates. However, we could interview a total of 452 respondents during the time frame for data collection.”* We have also revised the manuscript accordingly ***(Please see pages 5-6; lines 118-127).***

**Comment 2:** Sampling Technique: Though the authors made some attempts under study site and sampling size/data collection to explain the sampling technique used by this study, the explanation is still not clear. Therefore, kindly create a section titled “Sampling Technique” and explain step by step on how you selected your facilities and respondents. It appears you used a multistage sampling technique and you stated how you purposively selected the primary health centers used, however you did not explain how you allocated questionnaires to each of the centers and the number of questionnaires allocated. You did not also clearly state how and technique used in respondent recruitment over the study period or are these patients also selected using convenience sampling by the physician (a convenience sampling (non-probabilistic technique) may introduce bias into you study and reduce its soundness scientifically).

**Response:** Thanks for this comment. We have now provided sub-section with a detailed explanation of the sampling technique as follows ***(Please see pages 6-7; lines 129-153):***

***“Sampling technique***

*We collected cost data through surveys from both service providers and users to capture their perspectives.*

*Selection of health facility for survey*

*We purposively selected Chandpur district for this study, which has a total of seven UzHCs. These UzHCs were categorized into two groups based on the number of beds: 31-bedded (four) and 50-bedded (three) UzHCs. From each group, we selected two UzHCs based on their outpatient utilization rates—one with high utilization and one with low utilization. The outpatient utilization data for these facilities were extracted from the local health bulletin for the past three years (2018–2020) [19]. The selected 31-bedded UzHCs were Faridganj and Matlab North, while the selected 50-bedded UzHCs were Hajiganj and Kachua.*

*Patient selection for exit survey*

*To select patients for the exit survey, the most frequent outpatient diseases or symptoms were listed by consulting with the Upazila Health and Family Planning Officer and Medical Officers of the selected UzHCs. Each patient received a ticket that served as both a prescription and a record of their diagnosis, medication, and any diagnostic information provided by the physician. When patients presented with any of the selected conditions, the service providers informed our researchers, who systematically selected every second patient utilizing outpatient services for participation in the study.* *For patients under 18 years old or those unable to participate due to illness, an adult caregiver was interviewed instead. Using this approach, a total of 452 interviews were conducted, with 193 interviews from Hajiganj, 81 from Kachua, 85 from Faridganj, and 93 from Matlab (North) UzHCs.* *The number of patients interviewed varied across the UzHCs as per sample selection process.”*

**Comment on Results**

**Comment 3:** Table 4: It will be beneficial if an additional column is created to show percentage of cost paid by the patients out of the “Expected Medicine costs as per prescription´ for each diseases and total cost. This will show the percentage OOPE for each diseases and also cumulatively.

**Response:** Thank you for your suggestion. We have added two columns: (i) % share of the total estimated cost of medicines provided by UzHC and (ii) % share of the total estimated cost of medicines to be borne by patients. This now shows the percentage of estimated medicine costs covered by UzHC alongside the expected costs to be borne by patients ***(Please see page 16; Table 4).***

**Comment on Discussion**

**Comment 4:** Include the percentage of OOPE gotten in this study (as gotten in new table 4) in your discussion part from line 297 to 307. This will enable you make clearer comparison with

the “national-level study” value quoted on line 306.

**Response:** Thank you for this suggestion. We have incorporated the percentage of OOPE obtained in this study, as presented in the revised Table 4, into the discussion section *(****Please see page 18; lines 343-347).*** This addition allows for a clearer comparison with the national-level study value mentioned on line 345 *(previous line 306).*

**Comment on Conclusion**

**Comment 5:** The conclusion should also reflect your study outcome based on your objectives

**Response:** Thank you for your feedback. We have revised the conclusion to ensure it reflects the study's objectives and outcomes.

*“This study estimated the societal costs of outpatient healthcare services for common diseases in selected UzHCs of rural Bangladesh. Despite the government's obligation to provide free healthcare, a considerable gap exists between prescribed and available medications resulting into high OOPE on patients while seeking outpatient care from UzHCs. A need-based system for the supply of medicine is essential to ensure compliance with the treatment, especially for low-income population. Ensuring a reliable supply of essential medications and expanding diagnostic services at UzHCs will help reduce the financial burden, improve healthcare access and facilitate achieving UHC in Bangladesh.” (****Please see page 21; lines 408-415).***

**Response to Reviewer 2:**

--------------------------------

**Comment 1.** The present introduction states the purpose of the study and discusses how these findings can add value to the existing evidence. However, it is suggested to highlight a summary of previous research work to indicate gaps.

**Response:** Thank you for your valuable comments and suggestions. We have carefully reviewed the manuscript and emphasized the significance of the study. Additionally, we have cited previous research to highlight existing gaps in the literature (***Please see page 5; lines 99-107)***. However, we acknowledge that there are limited comparable studies in the context of Bangladesh, although we have incorporated all relevant existing studies.

**Comment 2.** Semi-structured questionnaires were administered; however, no reference was given for information about the number of questions, dimensions, and scoring of the questionnaires. Similarly, there was no reference to the validity and reliability of the research instrument.

**Response:** Thanks for this comment. We have now provided a clear statement on the study instruments as follows *“We employed a semi-structured questionnaire to gather data from health facilities and patients/caregivers. These questionnaires were developed based on existing literature and underwent review by experts, as well as by institutional and ethical review committees. Additionally, the questionnaire was translated into Bengali, contextualized for the Bangladeshi setting, piloted in similar environments, and finalized to ensure that respondents comprehended all the questions………”****(Please see pages 7-8; lines 155-183)***

**Comment 3.** The sampling design and methodology of the research is not quite clear.

**Response:** Thank you for your comment. We have thoroughly revised the methodology section, including a separate subsection for sample size calculation, sampling techniques, and data collection methods to enhance clarity and detail ***(Please see pages 5-11; lines 110-254).***

**Comment 4.** Language structure should be revised to reflect research rigor.

**Response:** Thank you for your suggestion. We have carefully revised the language and structure of the manuscript to enhance its clarity and rigor.
