# Supplementary material for: Costs of outpatient services at selected primary healthcare centers in Bangladesh: A cross-sectional study
Source: PLoS One. 2025 Jan 14;20(1):e0317317. doi: 10.1371/journal.pone.0317317 (PMC11731759; doi:10.1371/journal.pone.0317317)
Supplement: Attachment — Submitted filename: PLOS Review response on Cost 2.docx [file pone.0317317.s008.docx]

The author has made fair attempt at addressing the concerns raised.

**METHODOLOGY**

**Sample size**

The author has attended to the query under this section

**Sampling Technique**

The author has made fair attempt to make this clearer

**RESULTS**

**Table 4**

This has been modified as advised

**DISCUSSION**

A good attempt has been made in this regard.

**CONCLUSION**

Done as advised
