# Supplementary material for: Costs of outpatient services at selected primary healthcare centers in Bangladesh: A cross-sectional study
Source: PLoS One. 2025 Jan 14;20(1):e0317317. doi: 10.1371/journal.pone.0317317 (PMC11731759; doi:10.1371/journal.pone.0317317)
Supplement: Attachment — Submitted filename: Response to Reviewers.docx [file pone.0317317.s010.docx]

**Response to reviewer comments**

**Editorial comments**

**------------------------------**

Many thanks for the comments on the document. However, we have noticed that the comments were provided on the previous version of the manuscript thus most of the comments were addressed in the previous version.

**Comment**: Please change to "Introduction"

**Response**: We have now revised. ***(Please see page 3, line 55)***

**Comment:** PHC options like Union Health and Family Welfare Centers change like to “such as the”

**Response**: We have revised the background section. ***(Please see page 4, lines 75-77)***

**Comment:** Reference for the NHP 2011?

**Response**: Yes, the reference is for the National Health Policy 2011. ***(Please see page 4, line 83)***

**Comment**: What is OOPE? Please define with first use

**Response**: OOPE stands for out-of-pocket expenditure. We have abbreviated it in the introduction section, where it was first used. (***Please see page 3, line 60)***

**Comment:** This is not the description of the study site but the sampling technique. Please do a proper description of the surveyed area and move the other information to subheading "sampling technique" Additionally, I'd suggest the authors describe properly how these health facilities were selected. What sampling methods were used?

**Response**: Many thanks for the suggestions. We have revised the description of settings, sampling technique, and selection of healthcare facilities. (***Please see page 5, lines 113-115; page 7, lines 144-154)***

**Comment:** How did the authors arrive at this sample size? How were these patients selected from the health facilities?

**Response**: We have updated the sample size calculation in the latest version of the submitted manuscript. Please see the sample size sub-section. ***(Please see pages 5-6, lines 118-128)***

**Comment**: Why pre-selected symptom/diseases? It would be good to mentioned the criteria used to select these conditions. How were these diseases selected?

**Response**: Many thanks for the comment. We have now revised the section and added how we prepared the list of diseases. *“**To select patients for the exit survey, we initially prepared a list of the most frequent outpatient illness or symptoms. The list was prepared in consultation with the Upazila Health and Family Planning Officers and Medical Officers of the selected UzHCs. During the listing*

*, different facilities reported different illness as most frequent. However, from the collected list, we selected the illness those were reported as common in majority of the UzHCs to keep consistent with the type of illness in all UzHCs.”* ***(Please see page 7, lines 144-149)***

**Comment**: What is the source of this questionnaire? Please provide a reference if it has been published before or attach it as an appendix if it was designed by the authors? Give a more description of the study instrument. Also, give a detail of it validity and reliability.

**Response**: We have used a standard questionnaire for cost data collection following a previous study conducted by the authors. We have now included a line on the questionnaire and a reference. We have also added the following lines regarding the questionnaire “The questionnaire had several sections covering socioeconomic and demographic information of the respondents and households, details of illness, healthcare utilization and healthcare expenditure for the current illness episode, and patients’ opinions on healthcare services provided by the facility. ” ***(Please see page 7, lines 164-167)***

**Comment** “review of relevant documents” This was not mentioned as part of the study design

**Response:** Many thanks, we have now added a line in the study design section as follows. *“The quantitative method included a health facility survey and review of relevant costs document to estimate provider costs and a patient exit survey to assess the costs incurred by patients for utilizing outpatient services”* ***(Please see page 5, lines 114-115)***

**Comment**: Is there any inclusion and exclusion criteria for this study?

**Response**: The only inclusion criteria we considered that patients have to be diagnosed with the listed illness and respondents will have to be aged 18 and above which has been included under the sub-section “Patient selection for exit survey”. ***(Please see page 7, lines 151-156)***

**Comment:** Please remove the "e.t.c." Ditto for other part of the work. List all that was estimated.

**Response**: Thanks for the comment. We have revised it accordingly. ***(Please see page 9, lines 194-195)***

**Comment**: Human capital approach or friction cost method Which of them was used?

**Response**: We have used the human capital approach for costing. We have now revised this accordingly. ***(Please see page 10, line 222)***

**Comment**: I'd suggest that the USD rate is provided most especially, for the major cost average

**Response**: Many thanks for the suggestions. We have included the USD rate for the major cost average.

**Comment**: Table 1. Occupation (%) These didn't add up to 100. Confirm for the rest of the tables

**Response**: We have checked and revised the table*.* ***(Please see page 13, Table 1)***

**Comment:** Table 1. How was this (asset quintile) analysed? Please include in the methods

**Response**: Many thanks for the comment. We used principal component analysis to construct the asset quintiles based on the reported durable assets of the respondent. We have now added the following lines in the data analysis section under method. *“We classified the socioeconomic status of the respondents based on their reported possession of durable assets and applying principal component analysis (PCA). The PCA scores were generated using the possession of durable goods (e.g., mobile phones and televisions) [25]. Using the scores generated from PCA, the respondents were divided into five groups.”* ***(Please see page 11, lines 255-258)***

**Comment.** Table 4 and 5. It would be easier to follow if this could be arranged in either ascending or descending order

**Response:** Many thanks for the suggestion. We have now arranged the tables in descending order. ***(Please see page 17, Table 4; and page 18, Table 5)***

**Comment.** Table 5. CI for Provider costs (except medicine) in the malnutrition row?

**Response:** One limitation of our study is the variability in case observation across facilities during data collection. Although illness types were selected in consultation with service providers, certain cases were not observed in all facilities. For instance, the "Malnutrition" category, highlighted in your comment, was recorded in only one facility (Haziganj UzHC). Consequently, the CI for provider costs in this category could not be calculated. We have also highlighted this as one of the limitations of the study. ***(Please see page 22, lines 416-418)***

**Comment:** “a sizable number of patients (about 85%) still needed to purchase medicine and diagnostic services.” Is this part of the study findings? Kindly reference it if not.

**Response:** We have revised and removed this statement in the updated version.

**Comment**: “*It was estimated that the diagnostic services cost is the main driver of the expected OOPE followed by medicine costs which are justified by a study conducted by MoHFW [23].”* Additionally, this finding could be further compared with studies done in other parts of the world including sub-Saharan African such as

1. https://dx.doi.org/10.4314/jcmphc.v34i1.3,

2. https://www.ajol.info/index.php/jcmphc/article/view/139376,

3. doi: 10.4103/ijcm.ijcm_431_22 and

4. doi: 10.18311/jhsr/2018/21335

**Response:** We have compared this finding with studies conducted in other LMICs, including Ethiopia, the Democratic Republic of Congo, and Nigeria and included the suggested references. ***(Please see page 19, lines 350-352)***

**Comment:** *“Bridging this gap necessitates policy-level changes, including strengthening the existing rural healthcare system by guaranteeing a steady supply of medicines and increasing the number of essential diagnostic tests at UzHCs level.”* From here till the end of the paragraph are recommendations. Please move it and combine with the one at the conclusion section.

**Response:** Many thanks for the suggestion. We have revised it accordingly. ***(Please see page 22, lines 419-420)***

**Comment:** *“A fundamental shift towards a demand-driven allocation model in the healthcare financing strategy is required.”* The allocation of resources seems to be in-patients service demand driven already. I would suggest "taking into considering out-patient service demand in resource allocation" instead.

**Response:** Thanks for the suggestion, we have now revised the statement accordingly. However, the current resource allocation in UzHC is primarily based on number of facility beds and staffing levels, which do not adequately reflect the resources required as per the need of facility utilization by inpatients or outpatients. ***(Please see page 22, lines 419-420)***

**Comment:** Conclusion: *“...having potential side effects including medication non-adherence, antimicrobial resistance, and poor health outcomes.”* These are not study findings. Kindly delete.

**Response:** We have now revised and deleted this from the conclusion. ***(Please see page 22, lines 415-422)***

**Comment:** Conclusion: *“…a demand-driven”.* Please revise as suggested above.

**Response:** Many thanks for the suggestion. We have revised it accordingly. ***(Please see page 22, lines 419-420)***

**Reviewer comments**

**----------------------------**

Reviewer #2

**Comment:** Semi-structured questionnaires were administered; however, no reference was given for information about the number of questions, dimensions, and scoring of the questionnaires. Additionally, it is not clear if it is the same questionnaires/research instruments administered to both the facilities and the respondents considering the possible difference in information gathered from the different categories of respondents. Similarly, the validity and reliability of the research instrument is not clearly stated. Without this information, particularly the construct validity, the research is not replicable.

**Response**: We have used a standard questionnaire for cost data collection following a previous study conducted by the authors. We have now included a line on the questionnaire and a reference. (Reference: *Ahmed, S., de Broucker, G., Hasan, M. Z., Mehdi, G. G., Martin del Campo, J., Constenla, D., Patenaude, B., & Uddin, M. J. A.-B. & M. G. F. (2020). Cost of measles in children under 5 in Bangladesh (2017-18) (V2 ed.). Harvard Dataverse.* [*https://doi.org/doi:10.7910/DVN/ZXZEUY90*](https://doi.org/doi:10.7910/DVN/ZXZEUY90)*)* ***(Please see page 7, lines 164-167).*** We used separate questionnaires to collect data from health facilities and patients/caregivers. The details are elaborated in the "Study Instrument and Data Collection" subsection

***(Please see pages 7-8, lines 159-187)***
